# Evidence for current circulation of an ancient West Nile virus strain (NY99) in Brazil

**DOI:** 10.1590/0037-8682-0687-2020

**Published:** 2021-03-08

**Authors:** Márcio Junio Lima Siconelli, Daniel Macedo de Melo Jorge, Luiza Antunes de Castro-Jorge, Antônio Augusto Fonseca-Júnior, Mateus Laguardia Nascimento, Vitor Gonçalves Floriano, Fransérgio Rocha de Souza, Eudson Maia de Queiroz-Júnior, Marcelo Fernandes Camargos, Eliana Dea Lara Costa, Adolorata Aparecida Bianco Carvalho, Benedito Antonio Lopes da Fonseca

**Affiliations:** 1 Universidade São Paulo, Faculdade de Medicina de Ribeirão Preto, Departamento de Clínica Médica, Ribeirão Preto, SP, Brasil.; 2 Universidade São Paulo, Faculdade de Medicina de Ribeirão Preto, Departamento de Biologia Celular e Molecular, Ribeirão Preto, SP, Brasil.; 3 Ministério da Agricultura, Pecuária e Abastecimento, Laboratório Federal de Defesa Agropecuária de Minas Gerais, Pedro Leopoldo, MG, Brasil.; 4 Instituto de Defesa Agropecuária e Florestal do Estado do Espírito Santo, Ibiraçu, ES, Brasil.; 5 Agência de Defesa Agropecuária do Estado do Ceará, Boa Viagem, CE, Brasil.; 6 Ministério da Agricultura, Pecuária e Abastecimento, Departamento de Saúde Animal, Brasília, DF, Brasil.; 7 Universidade Estadual Paulista, Faculdade de Ciências Agrárias e Veterinárias, Departamento de Patologia, Reprodução e Saúde Única, Jaboticabal, SP, Brasil.

**Keywords:** West nile virus, NY99 strain, Horses

## Abstract

**INTRODUCTION:**

In Brazil, West Nile virus (WNV) was first detected, in 2018, in horses with neurological disease.

**AIM:**

We report the first case of WNV infection in a horse from Ceará state and the complete genome sequence of an isolate from Espírito Santo state. Both infections occurred in 2019.

**METHODS:**

WNV was isolated from the tissues of a horse with neurological signs in Espírito Santo and sequenced by MiSeq.

**RESULTS:**

Phylogenetic analysis revealed that the isolate belongs to lineage 1a, clustering with the NY99 strain, a strain that has not circulated in the USA since 2005.

**CONCLUSIONS:**

Our findings reinforce the hypothesis that WNV has been silently circulating in Brazil for many years.

The West Nile virus (WNV) is a zoonotic RNA virus belonging to the *Flaviviridae* and *Flavivirus* genera. It belongs to the Japanese encephalitis virus serocomplex that contains other viruses that cause neurological diseases in humans and animals, such as the Usutu, Saint Louis encephalitis, Murray Valley encephalitis, and Cacipacore viruses^1^ ^,^ ^2^. The classic WNV transmission network includes several birds as amplifier host/reservoirs (from several orders, but mainly from the order Passeriformes), and many mosquito species. Although WNV has been detected in many mosquito genera (i.e., *Aedes*, *Ochlerotatus*, *Anopheles*, and *Psorophora*) in North America, *Culex* mosquitoes are the main vectors involved in WNV transmission. These mosquitoes are involved in enzootic and epizootic transmissions, where horses and humans are accidental hosts and do not participate in the virus transmission network^3^. In the Americas, WNV was first detected in New York City in 1999, probably introduced from Israel^4^, and since then it has been detected in several American countries. Almost 20 years later, in 2018, the first molecular detections and viral isolations were performed in samples collected from a horse with a neurological disease, in a region from Espírito Santo State, Brazil^5^ ^-^ ^6^. Before 2018, serological evidence of WNV circulation was obtained from birds and horses from the Brazilian Amazon and Pantanal regions, and Paraíba state^3^ ^,^ ^7^ ^-^ ^9^. However, the first official human case was reported in 2014 in the Piauí state^10^.

In June 2019, two fatal cases of horses with neurological disease were reported to the Brazilian government, one from Espírito Santo (804_02_ES) and another from Ceará state (827_01_CE) (Figure 1). Clinical signs included neurological and locomotor disabilities, such as muscle tremors/rigidity, shaking of the head, weakness, ataxia, recumbency, hyperesthesia, limbs paresis, pedaling movements, and mydriasis. Central nervous system (CNS) samples were collected for diagnosis and laboratory investigation ruled out rabies, which was expected since both animals had been vaccinated recently. For further diagnostic investigation, samples were sent to the Federal Agricultural Defense Laboratory of Minas Gerais (LFDA/MG) of the Ministry of Agriculture, Livestock, and Supply (MAPA). WNV was detected using a RT-qPCR protocol recommended by the World Organization for Animal Health (OIE)^11^. Frozen CNS samples were sent to the Molecular Virology Laboratory of Ribeirão Preto Medical School - University of São Paulo (LVM-FMRP-USP) for further isolation and genome characterization.

**FIGURE 1:**
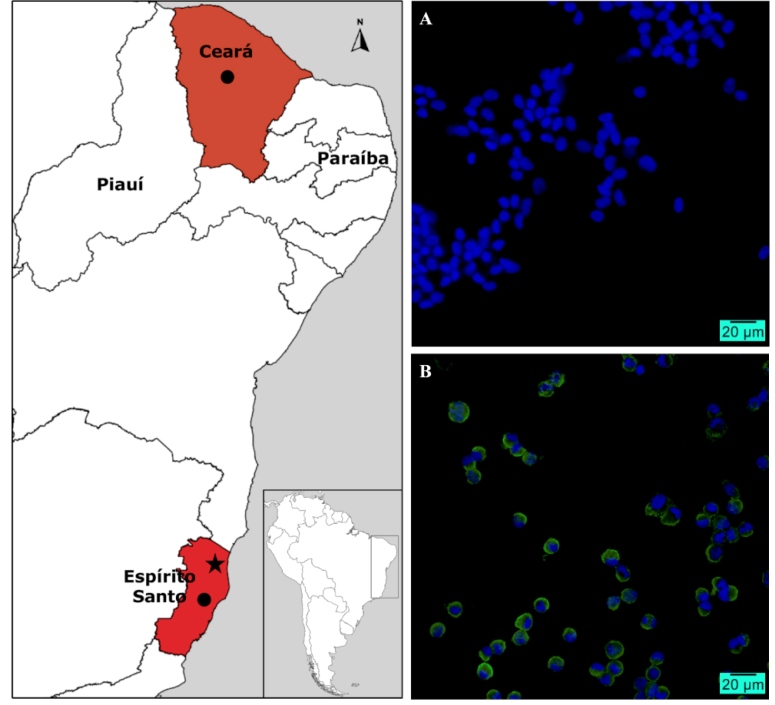
Map showing the states (in red) with molecular evidence of WNV circulation in horses with neurological disease. Black dots: municipalities of WNV occurrence (João Neiva in Espírito Santo (ES) and Boa Viagem in Ceará). Black star: municipality of São Mateus/ES where the first isolation and complete sequencing was done in 2018. Indirect immunofluorescence assay for the detection of West Nile virus antigens in VERO cells at 40X magnification. **(A)** Mock cells (uninfected cells were used as negative controls). **(B)** Infected cells after 48h post-inoculation with the 2nd passage of WNV isolate. Hyperimmune serum obtained from mice infected with WNV (NY99 reference strain) were used in both mock and infected cells. Blue: nuclei stained with DAPI Green: WNV antigen.

The samples were thawed on ice and 0.03-0.05 grams of either spinal cord (804_02_ES) or encephalon (827_01_CE) were manually macerated with a microcentrifuge pestle (Corning^®^) in DMEM (Hyclone^TM^) supplemented with 2% fetal bovine serum (FBS) and 2% antibiotic-antimycotic (Cultilab^TM^). The homogenate was clarified at 16,000 × g for 10 min at 4 °C and the supernatant was inoculated into VERO (CCL 81) and C6/36 (CRL 1660) cells. These cells were incubated at 37^o^C and 28^o^C, respectively, for 1h with gentle rocking every 10 min. The inoculum was then removed, the maintenance medium (2% FBS) was added to the monolayers, and the inoculated cells were incubated again, at 37 °C (VERO) and 28 °C (C6/36). After 5-6 days, cytopathic effects were clearly observed in VERO cells, and WNV was successfully isolated from 804_02_ES samples in both cell types. A second inoculation of both cells was performed to increase the virus titer.

Virus isolation was confirmed using RT-qPCR^11^ ^,^ ^12^ and an indirect immunofluorescence assay (IFA) with hyperimmune serum from mice infected with the WNV NY99 reference strain (Figure 1). The isolate (804_02_ES) and the clinical sample from Ceará state (827_01_CE) were analyzed using the MiSeq Illumina sequencing platform on the Life Sciences Core Facility (LMSeq) of São Paulo State University (UNESP/FCAV). 

We conducted a *de novo* analysis using a bioinformatics pipeline that focused on viruses. Software used on the analysis were FastQC version 0.11.8 (https://www.bioinformatics.babraham.ac.uk/projects/fastqc), Trimmomatic version 0.3.9 (http://www.usadellab.org/cms/?page=trimmomatic) and AfterQC version 0.9.7 (https://github.com/OpenGene/AfterQC). Virus assembly was mapped against the NY99 strain (GenBank accession: MH643887) to obtain a consensus sequence. The final consensus sequence of 804_02_ES was 10,893 nucleotides in length and an identity of 99.71% and 93.44%, for the nucleotide and protein, respectively. 

The 804_02_ES genome was aligned using MAFFT software (version 7.0) with a dataset obtained from NCBI and ViPR (https://www.viprbrc.org). A total of 116 WNV sequences worldwide (1953-2018) were selected. The dataset was edited and curated for use in the phylogenetic tree to maintain the entire ORF. The Maximum-Likelihood (ML) tree that was created using the IQ-TREE 2.0 software (http://www.iqtree.org) with automatic selection, was GTR+F+I+G4 the best nucleotide substitution model, with a support analysis of 10,000 replicates. The tree graph was edited using iTOL v.5 (https://itol.embl.de).

The 804_02_ES strain genomic sequence (GenBank accession: MT905060) was phylogenetically determined to belong to WNV lineage 1a (Figure 2) in accordance with the sequence obtained from the first isolate (isolated in 2018; GenBank accession: MH643887), diverging in only 32 nucleotides from each other^6^. The WNV MH643887 strain was also detected in samples from the Espírito Santo state (municipality of São Mateus), a region near the place where the sample yielding the isolate studied here was collected. TempEst v1.5.3 was used to determine the time of the most recent common ancestors (tMRCA) with a molecular clock, and to inspect and identify any inconsistencies in our sequence according to all databases of temporal structures.

**FIGURE 2:**
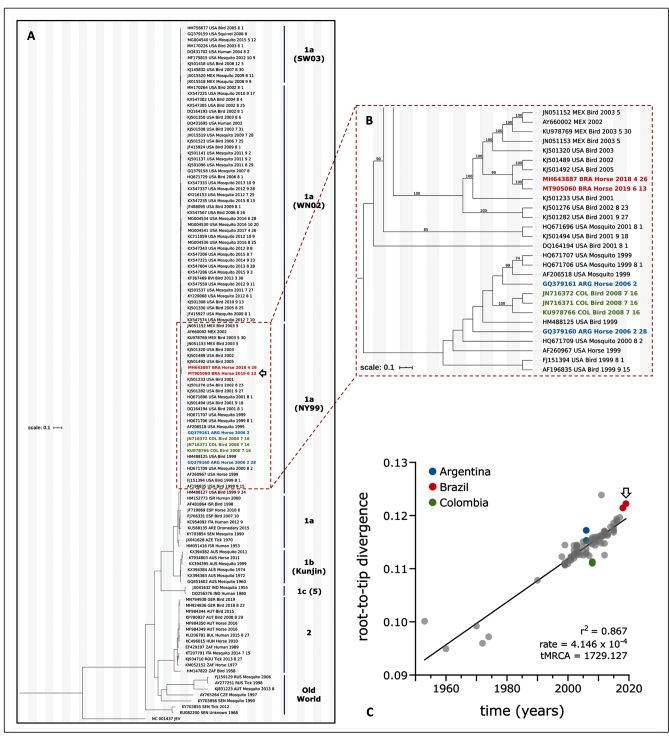
Maximum-likelihood (ML) phylogenetic reconstruction of WNV from 1953-2019. **(A)** a selection of 117 complete sequences from the global dataset were included in this analysis, presenting 0.1 substitutions per site. The GTR+F+I+G4 substitution model and support analysis of 10,000 replicates were used to obtain the phylogenetic tree; **(B)** dashed box highlighting South America (SA) strains, including the new isolate (MT905060), that cluster with NY99 strain, lineage 1a; only bootstrap values equal or bigger than 70% were shown; **(C)** root-to-tip analyses obtained with TempEst, using ML reconstruction obtained from 94 complete genomes from 1a and 1b lineages; graph correlation between time (years) and genetic divergence (substitutions per site) from the root of the tree (time to the Most Recent Common Ancestors, tMRCA) to the tips (sampled genomes) is shown; evolutionary slope rate: 4.146 x 10^-4^ substitutions/site/year; black arrow outline = new isolate. All colored strains belong to South America: Brazil (red), Argentina (blue), and Colombia (green). Old world lineages include: 3 (Rabensburg), 4a, 4b (9), 7 (Koutango), and 8.

According to the Brazilian Ministry of Health, WNV has been officially circulating in Brazil's northeast region since 2014^10^, although there has been serological evidence of WNV circulation since 2004/2008^3^. Our data confirm that WNV is circulating in Brazil, as we were able to isolate and obtain the complete genome sequence of a second isolate (GenBank accession: MT905060 (JNESEq804 strain)]. Although the NGS data were inconclusive regarding the complete genome sequence of WNV from 827_01_CE, WNV circulation in Ceará state was confirmed by the independent performance of RT-qPCR in both LFDA/MG (Ct 32.1) and LVM-FMRP-USP (Ct 27.6). In addition, a small portion of the amplicon was sequenced at the LFDA/MG, confirming the presence of WNV. Furthermore, our data suggests that the WNV NY99 strain has been circulating silently in Brazil and in South America (SA) for about two decades and this reinforces the view that WNV was probably introduced into the country many years before the first official report, probably between 2001 and 2005^6^. To date, all the sequenced SA WNV strains belong to the NY99 strain clade that had been displaced by two other strains [WN02 (env-V159A) and SW03 (NS4a-A85T)] in the USA, from 2001 to 2003. Of note, it was observed that after 2005, the NY99 strain disappeared completely from the USA^13^ strain.

Despite the evidence that WNV has been circulating in Brazil for some time, many questions still remain: (1) How was WNV introduced into Brazil and how has the NY99 signature been preserved until now? (2) Why was it not detected until-2014/2018? (3) Why has no other strain (WN02/SW03) been introduced (or detected) in the country? (4) Which mosquito and bird species are involved in the WNV transmission cycle in the Brazilian territory? (5) Is our most prevalent vector (*Culex quinquefasciatus*) capable of efficient transmission, similar to other *Culex* species prevalent in the USA? (6) Since WNV has been circulating in Brazil for many years, why has an outbreak of human infections not yet occurred? (7) Is this due to cross-protection induced by other endemic flavivirus infections (DENV, YFV, SLEV, or ZIKV)? 

At this moment, these questions cannot be answered because the available data are not enough to draw any conclusions, but it is almost certain that WNV was probably introduced into Brazil by bird migration or legal/illegal bird trade. Brazil is a tropical country with one of the most diverse fauna populations in the world. This could be a protective factor against an outbreak because every small part of the ecosystem needs to be taken into consideration in the complex the WNV transmission network to reduce viral activity^14^. To fill these gaps, it is necessary to implement a surveillance system that is sensitive enough to identify suspicious cases of neurological diseases in humans and animals, and to perform differential diagnoses for other virus species. Applying the One Health concept and the understanding that animals, in general, can be used as sentinels for disease activity will provide more data to understand the epidemiology of WNV in the Americas and make it possible to detect WNV circulation before it can cause an explosive outbreak, with many fatalities, as happened in the United States in 1999.

**Ethics:** Animal samples were collected by the Official Veterinary Service of each state and sent to the national reference laboratory, Federal Agricultural Defense Laboratory of Minas Gerais (LFDA/MG), from the Ministry of Agriculture, Livestock, and Supply (MAPA) to perform the diagnosis. Both animals died naturally. This study does not require approval by the Ethics Committees according to Brazilian normative resolution no. 30 of February 2, 2016, of the National Animal Experimentation Control Council (CONCEA).

IFA was performed using hyperimmune serum obtained by venipuncture from infected mice. The Ethics Committee on the Use of Animals (CEUA) from Ribeirão Preto Medical School, University of São Paulo, approved the study under process number 214/2019.
